# Neurofunctional Signature of Hyperfamiliarity for Unknown Faces

**DOI:** 10.1371/journal.pone.0129970

**Published:** 2015-07-08

**Authors:** Elisa Negro, Federico D’Agata, Paola Caroppo, Mario Coriasco, Federica Ferrio, Alessia Celeghin, Matteo Diano, Elisa Rubino, Beatrice de Gelder, Innocenzo Rainero, Lorenzo Pinessi, Marco Tamietto

**Affiliations:** 1 Department of Neuroscience, University of Torino, Torino, Italy; 2 Department of Neuroscience, Neuroradiology, University of Torino, Torino, Italy; 3 Department of Psychology, University of Torino, Torino, Italy; 4 Department of Medical and Clinical Psychology and CoRPS—Center of Research on Psychology in Somatic diseases—Tilburg University, Tilburg, The Netherlands; 5 Department of Cognitive Neuroscience, Maastricht University, Maastricht, The Netherlands; 6 Department of Experimental Psychology, University of Oxford, Oxford, United Kingdom; Zhejiang Key Laborotory for Research in Assesment of Cognitive Impairments, CHINA

## Abstract

Hyperfamiliarity for unknown faces is a rare selective disorder that consists of the disturbing and abnormal feeling of familiarity for unknown faces, while recognition of known faces is normal. In one such patient we investigated with a multimodal neuroimaging design the hitherto undescribed neural signature associated with hyperfamiliarity feelings. Behaviorally, signal detection methods revealed that the patient’s discrimination sensitivity between familiar and unfamiliar faces was significantly lower than that of matched controls, and her response criterion for familiarity decisions was significantly more liberal. At the neural level, while morphometric analysis and single-photon emission CT (SPECT) showed the atrophy and hypofunctioning of the left temporal regions, functional magnetic resonance imaging (fMRI) revealed that hyperfamiliarity feelings were selectively associated to enhanced activity in the right medial and inferior temporal cortices. We therefore characterize the neurofunctional signature of hyperfamiliarity for unknown faces as related to the loss of coordinated activity between the complementary face processing functions of the left and right temporal lobes.

## Introduction

Face recognition can be based on recollection or on familiarity. Recollection involves remembering specific contextual details concerning the where and when of past experiences, whereas familiarity supports face recognition without recovery of any episodic detail [1]. Discrimination between familiar and unfamiliar faces is essential for effective social interactions and involves a neural network distributed across hemispheres [2–5]. However, the specific role of the different neural structures underlying the discrimination between familiar and unfamiliar faces is still poorly understood, and neuroimaging studies on healthy participants have provided mixed findings [6–10]. The study of disturbances in the recognition of familiar faces can thus provide crucial missing evidence.

Acquired prosopagnosia, the inability to recognize familiar faces, is the best-known face recognition deficit and typically ensues from damage to the (right or bilateral) temporal lobes [11]. Conversely, hyperfamiliarity for unknown faces (HFF) is, in many respects, the reverse of prosopagnosia. In fact, it consists of the disturbing and abnormal feeling of familiarity for unknown faces, while recognition of known faces is normal [12]. HFF was originally described by Kraeplin [13] and has been reported more recently as a rare selective disorder (i.e., without concomitant basic visual perception deficits, delusion or confabulation episodes) in a few patients with focal lesions or epilepsy, usually involving the temporal lobes[3, 12, 14–18] and in one patient with Alzheimer Disease (AD) [19].

The current interpretation of the neurofunctional underpinnings of HFF postulates a pathophysiological imbalance between the complementary face processing functions of the left and right temporal lobes, in the context of interhemispheric inhibition [3, 12]. The notion is that impaired face processing in the damaged or hypofunctional left temporal lobe impedes face recognition based on individual facial features, and hampers recollection of specific semantic and episodic knowledge associated with unique identities. This leads, in turn, to the release and hyperactivity of the right temporal lobe functions that underlie subjective feelings of familiarity, personal relevance, and affective meaning, thereby inducing spurious recognition of unknown faces on the base of misdirected familiarity feelings. Hitherto, however, the neurofunctional signature of HFF has never been investigated with neuroimaging methods that are able to provide direct support for this hypothesis.

The study of patient GN, a 68-year-old woman with selective HFF, offered the unprecedented opportunity to investigate the neurofunctional correlates of HFF in a multimodal behavioral/neuroimaging experiment. We demonstrated atrophy and hypofunctioning of the left temporal regions both anatomically and functionally with morphometric analysis and single-photon emission CT (SPECT) scan, respectively. Conversely, functional magnetic resonance imaging (fMRI) revealed that HFF was selectively associated to enhanced activity in the right medial and inferior temporal cortices. We therefore provide compelling evidence that the neurofunctional signature of HFF is related to the loss of coordinated activity between the left and right temporal lobes.

## Methods and Materials

### Case Report

Patient GN is a 68-year-old right-handed woman without previous psychiatric or neurological episodes, and a normal seven-years education. She was admitted to our neurological clinic because of spontaneous complaints about mild episodic memory impairment and about a generalized and compelling feeling of familiarity and even intimacy for unknown people’s faces. This erroneous face familiarity recognition involved people met personally during daily life activities as well as people seen on television. HFF started about six months before admittance and gradually increased up to become severe and with direct consequences in daily life. In fact, HFF was spontaneous and immediate, and happened constantly throughout the day to the extent that for many (unknown) persons met or seen, GN engaged in effortful memory searching, trying to remember the circumstances, episodes and reasons that made the person’s face look familiar. The failure to recall such circumstance, and the stress related to it, leaded her to social detachment, as she stopped leaving the house or watching TV. By contrast, her ability to recognize truly familiar faces was unimpaired and correct recognition was associated with retrieval of the specific identity and correct name. GN never misidentified a person for another and never believed that people were disguised. Therefore, GN’s HFF appears as a selective disorder, qualitatively different from other misidentification syndromes, such as the Capgras (the pathological belief that a patient’s family member has been replaced by an identical impostor) or Fregoli syndrome (the delusional belief that different persons are in fact a single person in disguise) [20, 21].

Medical and neurological examination was entirely normal as well as standard laboratory blood tests. During the psychiatric examination the patient was cooperative, well groomed, oriented and with a euthymic mood. No evidence of a formal thought, language and mood disturbance or of other psychiatric symptoms was detected. She never reported epileptic disorders. GN was treated with donepezil (5 mg/die for the first month, and then 10 mg/die for other 5 months) with relieve from the mild memory impairment but not from the HFF, which persisted substantially unchanged until the second follow-up (six months after discharge) and then gradually abated until spontaneous remission.

A positive family history for dementia was reported, as GN’s mother had an early-onset AD. The presence of mild episodic memory deficits and the familiarity for dementia, suggested to further evaluating the possibility of a prodromal stage of AD. Therefore, additional metabolic and genetic analyses were carried out. There is indeed evidence that early stages of dementia are related to the increase of specific biomarkers in the cerebrospinal fluid (CSF) and to genetic factors. Analysis of the CSF (cells count: 1 element/mm^3^) was performed to assess the presence of biomarkers with high levels of sensitivity and specificity for amyloid load and tangles in the brain, which are characteristic of dementia. These markers are amyloid β1–42 (Aβ-42) (mean sensitivity: 86%; specificity: 90%), total tau (t-tau) (mean sensitivity: 81%; specificity: 90%), and phosphorylated-tau (p-tau) (mean sensitivity: 80%; specificity: 92%). Concentrations of the Aβ-42 were significantly reduced (298.40 pg/ml; normal values > 500 pg/ml), whereas concentrations of the t-tau were in the normal range (207 pg/ml; n.v. < 450 pg/ml) and concentrations of the p-tau were slightly increased (61.6 pg/ml; n.v. < 61 pg/ml). As far as the genetic factors related to dementia is concerned, GN has been found to carry the ε2–ε3 alleles of the apolipoprotein E (APOE), whereas higher risk to develop dementia and AD has been found in carriers of the ε4 allele of the (APOE) genotype. In consideration of the clinical history and of the assessment results, GN was diagnosed with atypical AD.

The neuropsychological examination took place during the first week after admittance, while the fMRI experiment and SPECT scan were performed three weeks after admittance.

### Controls

Twelve age-, gender-, and education-matched healthy controls with no previous history of neurologic or psychiatric disorders were enrolled in the study (mean age = 68.6 ± 3.06 years; education = 7 years).

### Stimuli and Procedure

**Fig 1:**
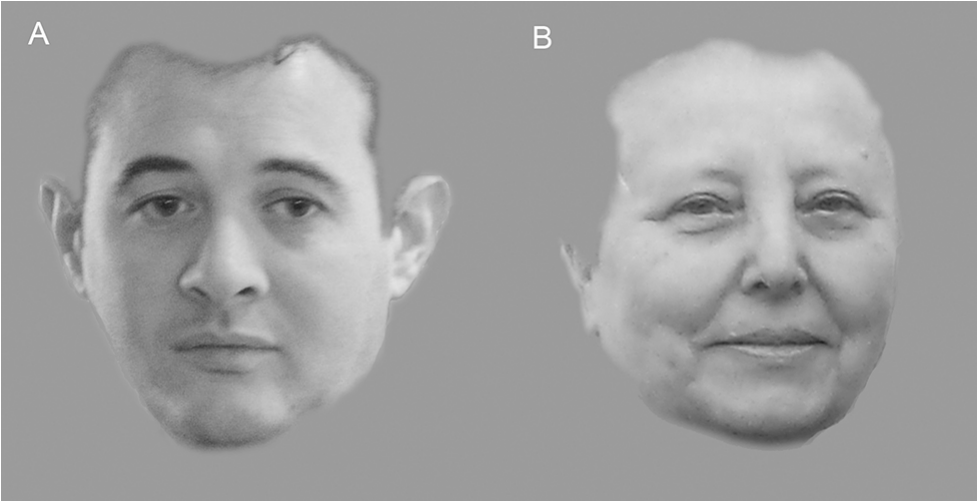
Examples of the faces used as stimuli. (A) Example of a male face personally known to patient GN. (B) Example of a female face unknown to patient GN.

The stimuli consisted of eighteen gray-scale digital pictures displaying faces of people personally known to the patient (i.e., family members, acquaintances, relatives and friends) and eighteen pictures of unknown faces, for a total of thirty-six pictures. All photographed volunteers underwent a protocol for the control of emotional neutrality (i.e., participants were asked to display no facial expression), gaze direction (looking straight at the camera) and facial angular view (faces in frontal pose). Half pictures were of males and half of female participants for each of the two categories. Unfamiliar faces were matched to familiar counterparts with respect to gender and approximate age. The pictures of personally familiar faces were taken by GN’s caregiver, unbeknownst to the patient, with a digital camera. To ensure that unfamiliar faces were indeed unknown to the patient, pictures of unfamiliar faces were taken among volunteers living outside the city area where GN always lived. All pictures were resized to sustain a visual angle of approximately 8° × 11°, the background, hair and neck was masked with uniform gray, and the mean luminance was adjusted to 15 cd/m^2^. The same procedure was used for the pictures of familiar faces presented to the age-, gender, and education-matched controls, whereas the same pictures of unknown persons displayed to patient GN were also presented to the controls (Fig 1).

The pictures were presented once each in a pseudo-random order for 4.6 sec. against a dark background on MRI compatible goggles with an average Inter-Stimulus Interval (ISI) of 6.9 sec (Resonace Technology Inc.). A central fixation cross was always present and a Fourier-transformed scrambled image with the same size, mean luminance and spatial frequency of the face images was used as baseline control for fMRI during rest periods. GN was instructed to judge whether the person presented was familiar or unfamiliar and to respond by button-press as quickly as possible. The right index and middle fingers of the dominant right hand were assigned to the familiar and unfamiliar responses, respectively. The identical procedure was used to test the controls, with the only exception that pictures were presented on a 21-in CRT monitor.

The present study was specifically approved by the local Bio-Ethical Committee of the University of Torino and performed in accordance with the ethical standards laid down in the 1964 Declaration of Helsinki. All participants, including patient GN, provided written informed consent also approved by the Bio-Ethical Committee of the University of Torino. In fact, as detailed in the “Case Report” section and formally assessed during the psychiatric and neurologic examination, GN was fully cooperative, oriented in space and time, and clearly able to read, understand and sign the consent form autonomously.

### Signal Detection Analysis

GN’s behavioral performance was analyzed with Signal Detection Theory (SDT) methods that consider not only correct recognition but also false alarms, which are particularly informative in the present case. In fact, SDT enables us to qualify familiarity decisions as depending on subject’s sensitivity to differences between familiar and unfamiliar faces (*d’*), as well as on subject’s response criterion (or bias), which is the tendency to favor a response independently of sensitivity (*c*) [22–25]. This distinction cannot be posed by simply comparing accuracy in sorting out familiar from unfamiliar faces, as normally done analyzing percentage of correct recognition, because this latter traditional approach does not consider false alarms and would reliably reflect familiarity sensitivity only assuming the absence of any response bias. According to SDT, the performance in yes/no tasks, like in the present case, is fully described by four parameters: hits, misses, correct rejections and false alarms. Hits refer to correct “yes” responses on signal trials; that is, on trials where a truly familiar face is displayed; whereas misses refer to incorrect “no” responses on signal trials. Correct rejections refer to correct “no” responses on noise trials; that is, on trials where the unfamiliar faces are displayed; whereas false alarms refer to incorrect “yes” responses on noise trials (i.e., hyperfamiliarity).

Familiarity sensitivity has been measured as *d*_*a*_, a variant of *d’* that is appropriate in case of unequal variance between distributions and is numerically equal to *d’* in the case of equal variance. The response criterion has been measured as *c*_*a*_, a variant of *c* that is appropriate in case of unequal variance between distributions and is numerically equal to *c* in the case of equal variance [26]. Finally, GN’s values on *d*_*a*_ and *c*_*a*_ have been compared with the corresponding mean values obtained in the control group by a series of one-sample *t*-tests to assess statistically significant differences. The method proposed by Crawford and Howell [27] has been applied to the *t*-test results, so as to correct for possible deviations from normality in small samples of controls by considering control sample mean values as statistics rather than as parameters (Singlims software).

### SPECT Data Acquisition and Analysis

GN was kept in a resting state for 20 min, lying supine on a stretcher in a quiet and dim-lit room with her eyes closed and ears plugged. Then, she was injected with 900 MBq of Tc-99m-HMPAO. Brain SPECT images were obtained using a dual-head gamma camera (BrightView, Philips) equipped with fan beam collimators. Each scan was obtained performing 120 frames of 128 × 128 pixels over a 360° orbit with a total acquisition time of about 30 min. Trans-axial, sagittal, and coronal images were reconstructed using a filtered back-projection algorithm with a Butterworth filter, and uniform attenuation correction was performed applying a first order Chang algorithm. The tomographic resolution of the system was of about 10 mm.

Quantitative analysis was performed with JetPack 1.0 by comparing with a series of paired-sample *t*-tests the perfusion in four ROI pairs, each encompassing the left and right frontal, temporal, parietal and occipital lobes of a composite image created by the cumulative counts of eight slices. Perfusion in the different ROIs was expressed as percentages, applying to the counts within each ROI in the left hemisphere the formula: left counts / left + right counts; and to each ROI in the right hemisphere the formula: right counts / left + right counts.

In addition, an interhemispheric asymmetry index (*AI*) was calculated for each pair of lobes (*l*), so as to enable direct comparison with previously reported data on perfusion in normal control subjects [28, 29]. It is indeed well established that brain perfusion and metabolism, as assessed with SPECT or PET, is highly symmetrical across hemispheres and lobes, and interhemispheric asymmetry is taken as index of brain pathology and suggested as a diagnostic feature for AD [28, 29]. Accordingly, the *AI*_*l*_ for a given pair of lobes was calculated as follows:

AIl=(%pLl-%pRl)*2(%pLl+%pRl)

where *%pL*_*l*_ is the percentage of perfusion (*p*) in the left hemisphere (*L*) for a specific lobe (_*l*_), and *%pR*_*l*_ is the percentage of perfusion for the same lobe of the right hemisphere (*R*). Therefore, the index ranges from -2 to +2, with negative values indicating interhemispheric imbalance due to relative hypoperfusion in the corresponding lobe of the left hemisphere, and positive values indicating asymmetry due to relative right-hemisphere hypoperfusion, whereas 0 denotes perfect interhemispheric perfusion symmetry.

### (f)MRI Data Acquisition

Data acquisition was performed on a 1.5-T Philips Achieva with an 8 channels Sense high-field high-resolution head coil optimized for functional imaging. Functional T2*-weighted images were acquired using echoplanar (EPI) sequences (TR = 2300 msec.; TE = 40 msec.; flip angle = 90°; acquisition matrix = 128 x 128; FoV = 230 mm.). A total of 218 volumes were acquired. Each volume consisted of 30 axial slices parallel to the anterior-posterior commissure line and covering the whole brain; the slice thickness was 4 mm with no gap. Three scans were added at the beginning of functional scanning and the data discarded to reach a steady-state magnetization before acquisition of the experimental data.

In the same session, a set of three-dimensional high-resolution T1-weighted structural image was acquired. This data set was acquired using a Fast Field Echo (FFE) sequence (TR = 7 msec.; TE = 3 msec.; flip angle = 8°; acquisition matrix = 256 x 256; FoV = 256 mm.). The set consisted of 190 sagittal contiguous images covering the whole brain. The in-plane resolution was 1 x 1 mm and slice thickness was 1 mm (isotropic 1mm^3^ voxel). The whole session lasted about 30 min.

### Sulcal Depth Analysis

Sulcal depth maps have been created for patient GN and for the twelve controls in BrainVoyager QX 2.1 (Brain Innovation, Maastricht, NL). T1-weighted structural images of each participant underwent two voxel-wise segmentations based on two intensity values. The first segmentation defined the outer contours of the brain without extensions into the sulci, whereas the second segmentation defined the white matter/gray matter boundary. A distance transform was then calculated resulting in values at each voxel that measure the shortest distance in mm. from that voxel to the white matter.

Three-dimensional surface maps of GN’s brain and of the brains of the twelve controls were created, and patches-of-interests (POI) were defined anatomically and individually for each hemisphere and participant so as to include all six sulci present in, or bordering with, the temporal lobes. These sulci are the lateral sulcus, the inferior temporal sulcus (both further divided in its anterior and posterior parts), the superior temporal sulcus, the anterior transverse collateral sulcus, the lateral occipito-temporal sulcus, and the collateral sulcus. A series of one sample *t*-tests were used for statistical comparisons of the mean sulcal depth in the control group for each sulcus and hemisphere with the sulcal depth in the corresponding sulci of patient GN, resulting in a total of eight comparisons for the left and eight for the right hemisphere. The same correction proposed by Crawford and Howell [27] was applied also to this series of *t*-tests.

### fMRI Data Analysis

BOLD imaging data were analyzed using the Statistical Parametric Mapping package (SPM8, http:/www.fil.ion.ucl.ac.uk/spm), in MATLAB 7.5 environment (Math Works Inc, Natick, MA, USA). Functional images were pre-processed as follows: i) 3-D motion correction was applied using a trilinear interpolation algorithm, all volumes were spatially aligned to the mean volume by rigid body transformations; ii) the structural images were co-registered with functional data to maximize the mutual information; iii) structural co-registered images were segmented in gray and white partitions to compute the MNI template normalization parameters; iv) structural and functional images were normalized in the MNI space; v) spatial smoothing was performed using an isotropic Gaussian kernel of 8 mm FWHM; vi) high-pass temporal filtering (cut-off 0.008 Hz) was used to remove low frequency artifacts.

Statistical analyses were carried out with General Linear Models, using square wave functions convolved with a canonical hemodynamic response function (HRF), and its temporal and dispersion derivatives were used to model the responses to each stimulus condition (familiar and unfamiliar regressors). Derivatives constitute a better model of the brain responses insofar as the neural events are fitted with 3 basis functions: the canonical HRF, its temporal partial derivative that adjusts for the onset latency of the peak response, and its dispersion partial derivative that adjust for the duration of the peak response [30]. Six movement parameters (3 translation, 3 rotation), estimated from realignment preprocessing step, were added to the GLM as covariate of non-interest to remove artifactual movement-related activations.

The spatially preprocessed images were analyzed with a single-subject fixed effects model and weighted *t*-contrasts were computed. A threshold of *q* < 0.05 corrected for false discovery rate (FDR) was applied to the final statistical maps of the contrasts (i.e., the false positive rate was controlled to be lower than 5%,), and clusters with less than 40 voxels were discarded (Ke > 40). Therefore, we choose a conservative threshold to minimize the risk of false positives.

## Results

### Neuropsychological Assessment

GN underwent an extensive neuropsychological evaluation of the general cognitive functions [31], reasoning abilities [32], executive functions [32, 33], language [34, 35], praxis [32, 34, 35], memory [36–38], visuo-spatial and perceptual functions [39–41]. Results showed that cognitive functions were entirely normal, including visual face perception, and documented only a mild impairment in verbal memory, which is an ability typically associated with the left temporal lobe functions [42] (Table 1).

**Table 1:** Patient GN’s Neuropsychological Assessment.

Tests	Patient GN^a^	Normative cut-off
General Cognitive Functions		
MMSE^b^	30.27	≥ 24,00
Reasoning		
Abstract thinking	30.90	≥ 18.96
Mental calculation	04.00	≥ 02.00
Executive Functions		
Trail Making test A and B	03.00	≥ 02.00
FAB^c^	19.97	≥ 12,03
Language		
Token test	32.75	≥ 26.50
Verbal letter fluency	36.60	≥ 17.35
Verbal category fluency	18.00	≥ 07.25
Praxis		
Constructional praxis	04.00	≥ 02.00
Ideational praxis	04.00	≥ 02.00
Ideomotor praxis	04.00	≥ 02.00
Memory		
Corsi Block Test	04.50	≥ 03.50
Digit span forward	05.50	≥ 03.75
Memory for prose	01.00*	≥ 02.00
Rey word list learning(immediate recall)	40.10	≥ 28.53
Rey word list learning(delayed recall)	05.40	≥ 04.69
Visuo-spatial and perceptual functions		
Benton facial recognition test	45.00	≥ 39.00
Clock drawing test	01.00	≤ 03,00
Street completion test	04.00	≥ 02.00

^a^All scores are corrected for age and education.

^b^MMSE = Mini Mental State Evaluation.

^c^FAB = Frontal Assessment Battery.

* = Score under normal limits.

### Structural and Functional Brain Abnormalities

**Fig 2:**
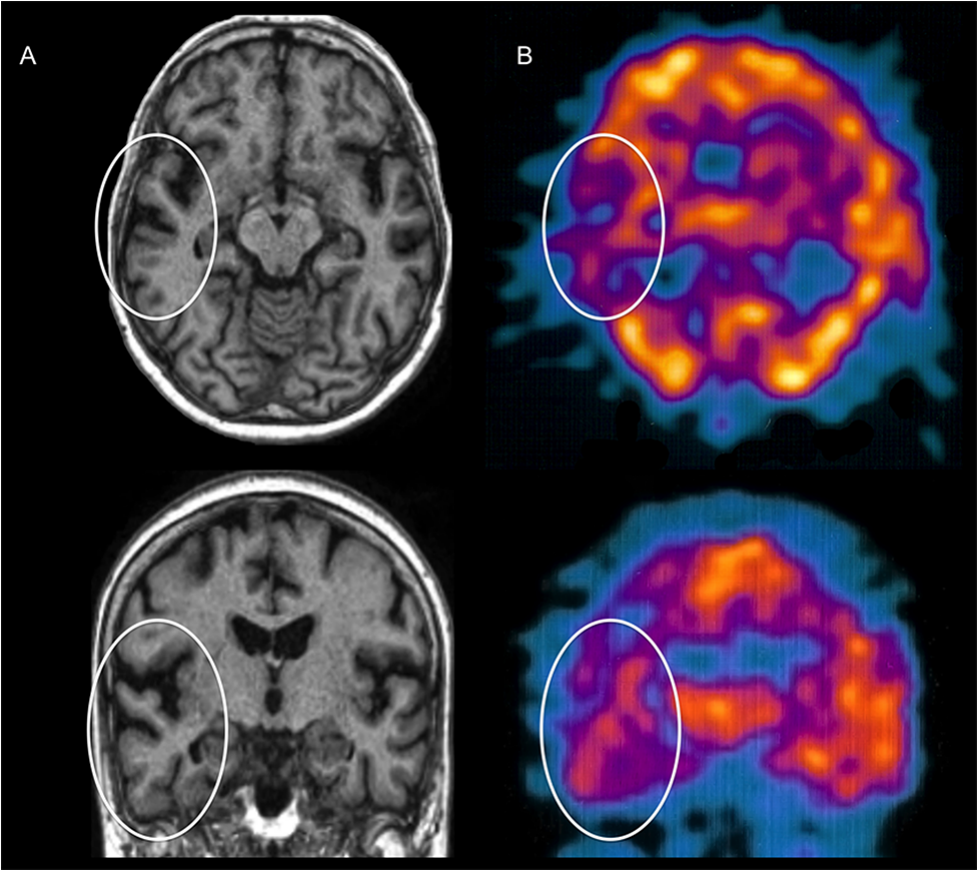
Anatomical MRI and SPECT of Patient’s GN Brain. (A) T1-weighted MRI transversal and coronal images showing atrophy in the left temporal lobe (white circles). (B) SPECT transversal and coronal images showing hypoperfusion in the left tempo-parietal region (white circles).

A structural T1-weighted MRI showed left temporal lobe atrophy with deepening and widening of the temporal sulci (Fig 2A). Consistent with the anatomical findings, a 99mTc CERETEC single-photon emission CT (SPECT) scan showed a decreased uptake in the left temporo-parietal region indicative of hypoperfusion (Fig 2B).

**Fig 3:**
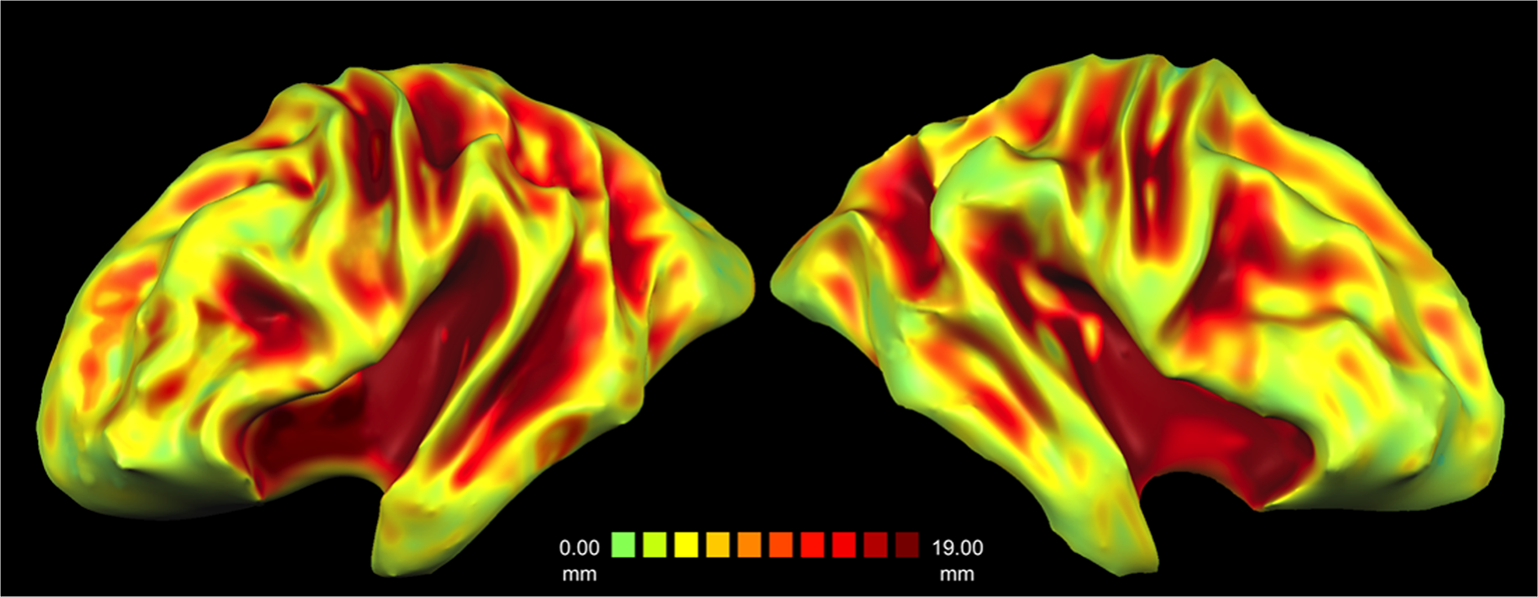
Sulcal Depth of Patient’s GN Brain. (A). Three-dimensional reconstruction of the sulcal depth in GN’s brain, from surface and shallow depth values (green/yellow) to deep values (orange/red). Note the deepening and widening of the temporal sulci in the left compared to right hemisphere as outlined by expanded red areas in lateral sulcus and in the STS.

**Fig 4:**
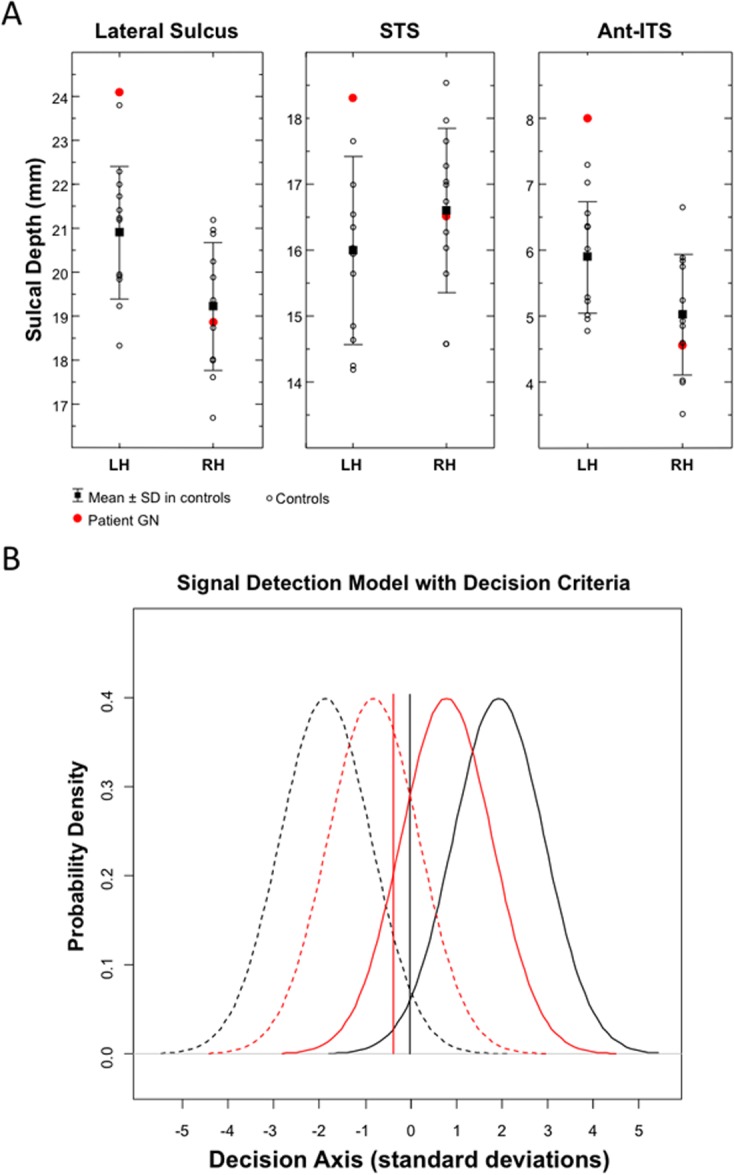
Sulcal Depth Values and Signal Detection Response Parameters. (A) Sulcal depth values for the lateral sulcus, STS and the anterior part of the ITS in the left (LH) and right hemisphere (RH) of patient GN and of the twelve age-, gender, and education-matched controls. (B) Signal detection parameters in patient GN (red lines) and averaged parameters in the twelve controls (black lines). High discrimination sensitivity between familiar and unfamiliar faces (*d*_*a*_) is graphically represented by reduced overlapping between signal distributions (familiarity) (continuous lines) and noise distributions (unfamiliarity) (dashed lines). Response bias (*c*_*a*_) is represented by the vertical lines, with negative values indicating a loose response criterion (i.e., a tendency to favour familiarity responses).

Morphometric quantification with sulcal depth analysis found that the posterior segment of the lateral sulcus, the superior temporal sulcus (STS) and the anterior part of the inferior temporal sulcus (ITS) in the left hemisphere of GN were significantly deeper and wider than the corresponding sulci of the twelve age-, gender-, and education-matched controls (*t*_11_ ≥ 2.025, *p* ≤ 0.034 one-tailed), whereas there was no significant difference for the other sulci in either hemispheres (*t*_11_ ≤ 0.521, *p* ≥ 0.3) (Figs 3 and 4A).

The percentage of perfusion in the left and right frontal lobes was, respectively, 52% and 48%, in the left and right temporal lobes was 45% and 56%, in the left and right parietal lobes was 46% and 54%, and, finally, it was 50% in the left and right occipital lobes alike. The hypoperfusion in the left temporal and parietal lobes was statistically significant when compared to the perfusion in the corresponding lobes on the right hemisphere (*t*_7_ = 2.966, *p* < 0.021 and *t*_7_ = 2.5679, *p* < 0.035, respectively), whereas the difference between the left and right frontal and occipital lobes was not significant.

Similarly, the *AI* index for the temporal and parietal lobes was − 0.22 and − 0.16, respectively, whereas the same index for the frontal and occipital lobes (0.08 and 0, respectively) demonstrated an almost perfect interhemispheric symmetry in perfusion. Therefore, the inter-hemispheric asymmetry due to hypoperfusion in the temporal and parietal lobes of the left hemisphere was between 13- to 18-fold higher than what reported with the same index in the literature on healthy subjects (mean = − 0.012, SD = 0.08) [29].

### Behavioral and Signal Detection Results

GN was almost faultless in recognizing truly familiar faces, but she mistakenly judged 1/3 of the unknown faces as familiar, an error rate more than 35-fold higher than that of matched controls (Table 2). GN’s discrimination sensitivity (*d*_*a*_) was significantly lower than that of matched controls (*t*_11_ = 15.9, *p* < 0.0001) and her response criterion (*c*_*a*_) for familiarity decisions was significantly more liberal (*t*_11_ = 5.378, *p* = 0.0002), revealing a marked bias toward considering unknown faces as familiar (Fig 4B).

**Table 2:** Recognition of Face Familiarity in Patient GN and in the Twelve Age-, Gender- and Education-matched Controls.

			**Face Stimuli**	
**Subjects**			***Familiar***	***Unfamiliar***
**Patient GN**				
	***Response***			
		***Familiar***	16/18 (88.9%)	6/18 (33.3%)
		***Unfamiliar***	2/18 (11.1%)	12/18 (66.7%)
	*Signal Detection*			
	Discrimination (*d*_*a*_)	1.65		
	Response bias (*c*_*a*_)	-0.39		
			**Face Stimuli**	
			***Familiar***	***Unfamiliar***
**Controls**				
	***Response Mean ± SD***			
		***Familiar***	18 ± 0 (100%)	0.17 ± 0.39 (0.94%)
		***Unfamiliar***	0 ± 0 (0%)	17.8 ± 0.39 (98.88%)
	*Signal Detection*			
	Discrimination (*d*_*a*_)	3.798 ± 0.13		
	Response bias (*c*_*a*_)	-0.027 ± 0.06		

### fMRI Results

**Fig 5:**
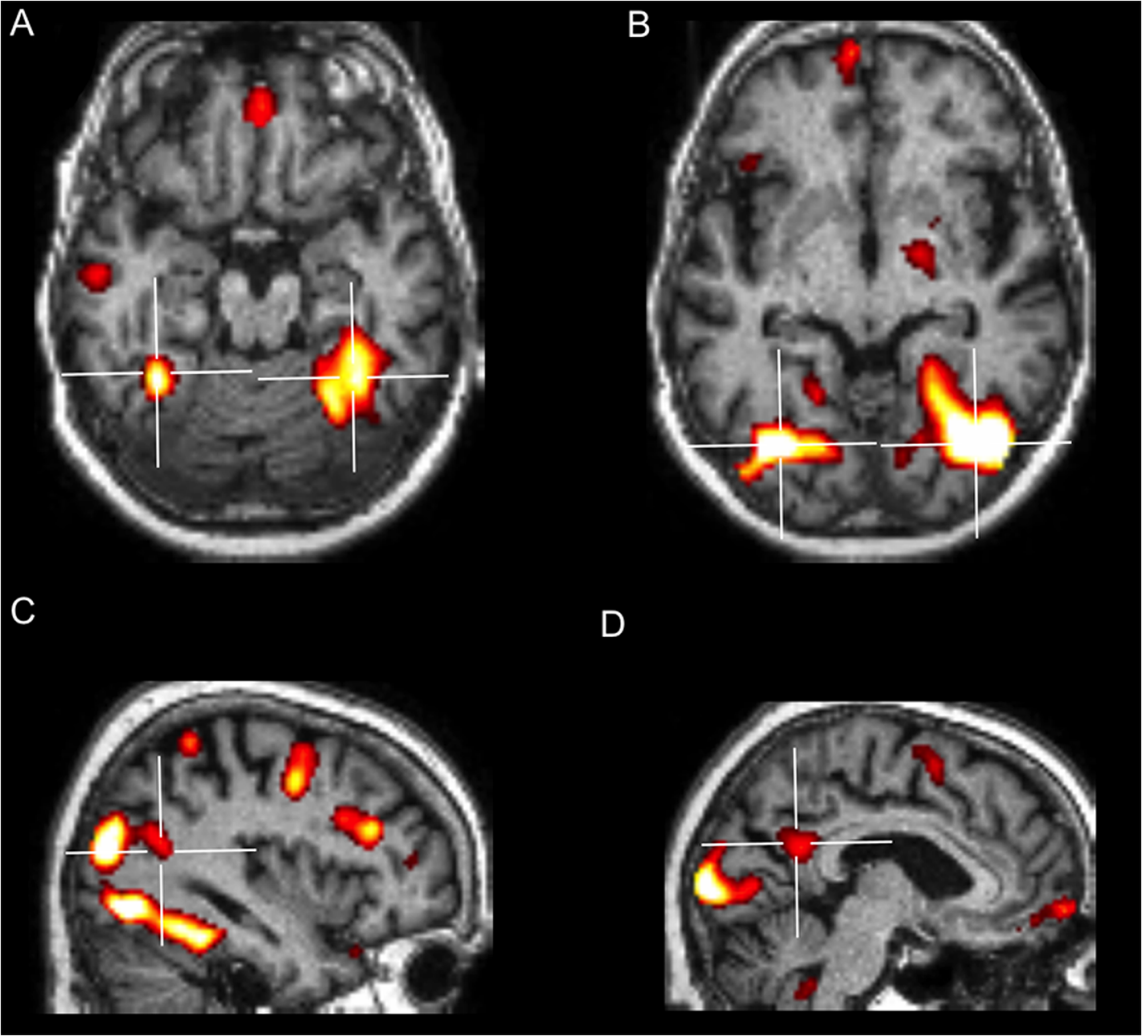
Brain areas significantly activated by the presentation of faces (known and unknown faces pooled together > baseline activity). (A) Activations in the left and right FFA. (B) Activations in the left and right OFA. (C) Activation in the right pSTS. (D) Activation in the PCUN/pCING.

A first preliminary analysis compared activations for faces (known and unknown pooled together) with baseline activity generated by the presentation of the scrambled image. This contrast was performed to verify whether, independently from familiarity, GN showed activations in the areas typically known respond to face in healthy subjects, as when a face localizer is used. Results showed a widespread neural network characteristic of normal face perception, which included brain structures composing the core as well as the extended face processing system [6, 43] (Fig 5 and Table 3).

**Table 3:** Areas Activated in Patient GN for Faces (Known and Unknown Pooled Together) vs. scrambled face baseline.

		Brain Area	H	BA	Ke	Ze	MNI Coordinates		
							*X*	*Y*	*Z*
**Face Processing System**									
	Core System								
		FFA	L	37	246	∞	-36	-50	-18
		FFA	R	37	367	∞	36	-42	-20
		OFA	L	19	107	∞	-38	-74	-8
		OFA	R	19	337	∞	44	-68	-8
		pSTS	R	39	45	4.96	38	-52	16
	Extended System (personal knowledge)								
		APC/MFG	L	10	423	5.37	-6	60	-4
		APC/MFG	R	10	=	5.05	2	46	-18
		TPJ	R	39	233	4.83	42	-70	20
		aTemp/MTG	L	20	180	5.06	-56	-12	-16
		aTemp/TP	R	38	105	4.46	42	20	-24
		PCUN/pCING	L	30	395	4.97	-6	-52	20
		PCUN/pCING	R	30	=	4.29	2	-48	24
	Extended System (emotion)								
		AMG	R	-	23	3.39	18	-8	-16
		INS	L	48	231	4.20	-42	26	-4
		INS	R	48	60	3.5	44	-12	-2
**Visual system**									
		CS	L	17	8088	∞	-10	-92	2
		CS	R	17	=	∞	12	-82	2
		MOG	L	18	=	∞	-18	-88	14
		PULV	R	-	86	4.89	20	-30	-2
**Motor and cognitive system**									
		MI/PCG	L	6	378	7.33	-44	-6	54
		MI/PCG	R	6	614	6.94	38	-2	42
		SMA	L	32	100	4.34	6	16	44
		GP	R	-	410	4.50	18	-6	-6
		CRBL	L	-	231	4.16	-2	-48	-40
		FP/SFG	R	10	246	5.84	16	66	10
		dlPFC/MFG	L	44	307	5.80	-48	26	32
		dlPFC/IFG	R	45	1643	∞	40	30	24
		vlPFC/IFG	L	45	51	4.07	-58	20	10
		SPL	R	40	167	5.46	36	-46	48

***Abbreviations*:** AMG = amygdala; APC = anterior paracingulate cortex; aTemp = anterior temporal cortex; BA = Brodmann area; CRBL = cerebellum; CS = calcarine sulcus; dlPFC = dorso-lateral prefrontal cortex; FFA = fusiform face area; FP = frontal pole; GP = globus pallidus; H = hemisphere; Ke = cluster equivalent voxel; IFG = inferior frontal gyrus; INS = insula; MFG = middle frontal gyrus; MI = primary motor cortex; MOG = middle occipital gyrus; MTG = middle temporal gyrus; OFA = occipital face area; PCG = precentral gyrus; pCING = posterior cingulate cortex; PCUN = precuneus; PULV = pulvinar; SFG = superior frontal gyrus; SMA = supplementary motor area; SPL = superior parietal lobule; pSTS = posterior superior temporal sulcus; TP = temporal pole; TPJ = temporo-parietal junction; vlPFC = ventro-lateral prefrontal cortex; Ze = equivalent Z.

Additionally, to further qualify the activations in visual areas composing the core face processing system, we compared by superimposition the locations of patient GN’s activations in the temporal and occipital lobes with functionally-defined face-selective regions, as reported by Julian and co-authors, who used a localizer design on a large group of healthy subjects [44] (downloaded from http://web.mit.edu/bcs/nklab/GSS.shtml). The regions, or face parcels, that were activated systematically across subjects in the study by Julian and collaborators [44] are the fusiform face area (FFA; including 2771 voxels in the left and 9706 voxels in the right hemisphere), the occipital face area (OFA; with 2145 voxels in the left and 7116 voxels in the right hemisphere) and STS (7651 voxels in the left and 9706 voxels in the right hemisphere). GN’s activations overlapped substantially with face parcels activation maps on all 3 areas composing the core system for face processing. Specifically, all 246 voxels significantly active in GN’s left FFA were also included in the left FFA localized by Julian at al. [44]. Of the 376 voxels in GN’s right FFA, 317 (84.3%) overlapped with those found in the localizer. As far as OFA is concerned, 99 of the 107 voxels activated in GN’s left hemisphere (92.5%) corresponded to the independent face-localizer, while there was a complete overlap for the 337 voxels in the right hemisphere. Finally, 32 of the 45 voxels found active in GN’s right STS (71.1%) were comprised in the STS parcel identified by Julian et al [44]. These results document that the neural response to face images in GN is substantially equivalent to the activations found in healthy observers when a face localizer is used, and are thus in keeping with the neuropsychological examination that also revealed no basic deficit of face perception in patient GN.

**Fig 6:**
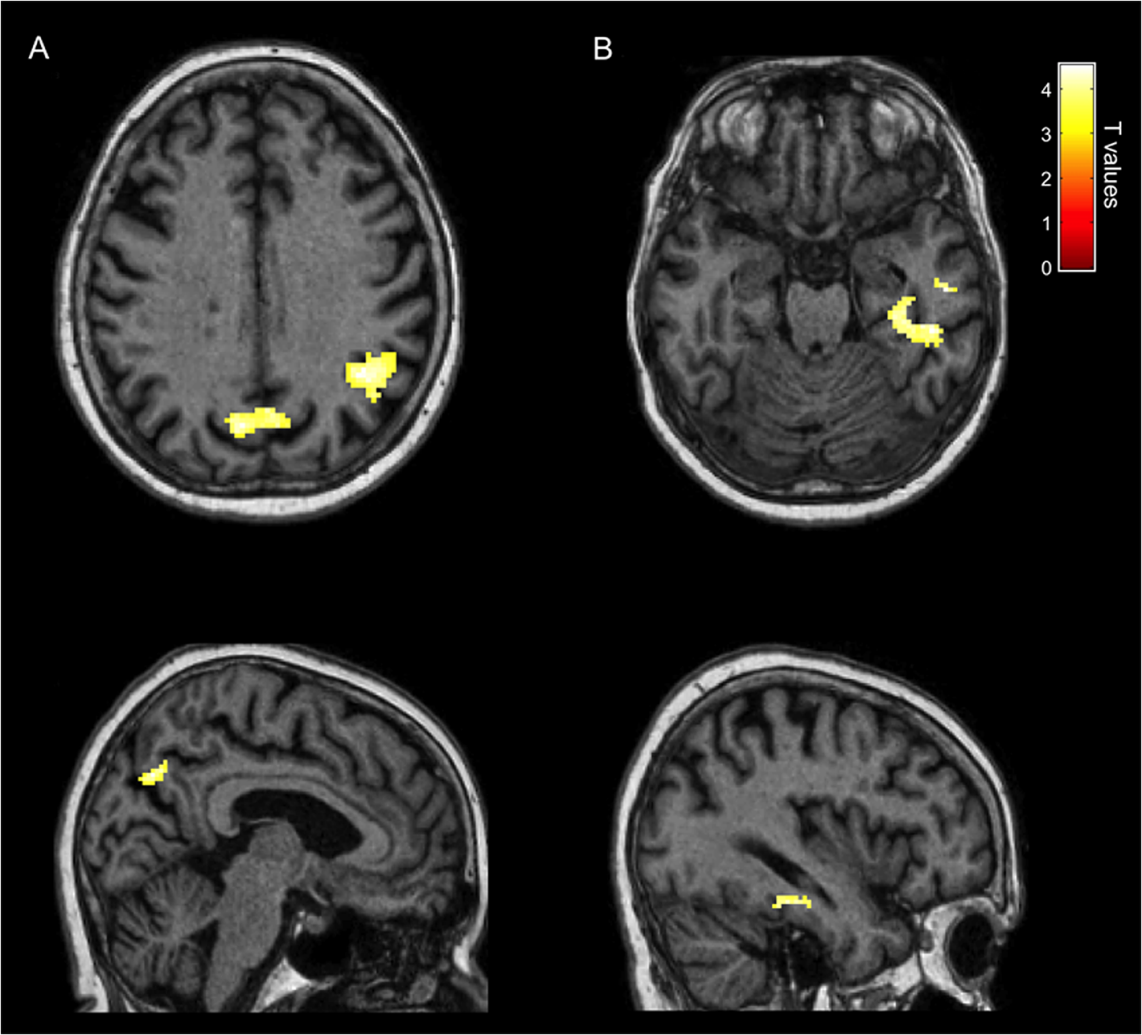
Neurofunctional Signature of Correct Familiarity and of Hyperfamiliarity for Faces. (A). Neurofunctional signature of correct face familiarity recognition displaying highly significant activations in the right pSTS/TPJ and in the precuneus bilaterally (known faces correctly recognized as familiar > unknown faces correctly recognized as unfamiliar). (B). Neurofunctional signature of HFF displaying highly significant activations in the right medial and lateral inferior temporal cortex, including the parahippocampal and the fusiform gyrus (unknown faces wrongly recognized as familiar > unknown faces correctly recognized as unfamiliar).

A second contrast analyzed the neural structures involved in the proper judgment of familiarity by comparing correctly recognized familiar faces with unknown faces correctly judged as unfamiliar. This contrast revealed bilateral activity in the precuneus and in the posterior part of the right STS (pSTS), including the temporo-parietal junction (TPJ) (Fig 6A and Table 4). Notably, these areas have been previously associated to correct familiarity judgments for personally familiar faces in healthy subjects [6, 45].

**Table 4:** Areas Activated in Patient GN for Correct Familiarity Recognition (known faces correctly recognized as familiar > unknown faces correctly recognized as unfamiliar).

Brain Area	H	BA	Ke	Ze	MNI Coordinates		
					*X*	*Y*	*Z*
pSTS/TPJ	R	39	178	4.48	49	-55	27
PCUN	L	7	55	4.37	-6	-74	38
PCUN	R	7	51	4.24	4	-70	36

Lastly, and most importantly, we investigated the neural activity selectively associated to HFF by comparing the neural response to unknown faces wrongly recognized as familiar with the activity associated to unknown faces correctly judged as unfamiliar. This comparison revealed significantly enhanced activity in the right medial and lateral inferior temporal lobe, including the fusiform gyrus (FG) and the parahippocampal gyrus (PHG) (Fig 6B and Table 5). No region was significantly deactivated in this comparison.

**Table 5:** Areas Activated in Patient GN for HFF (unknown faces wrongly recognized as familiar > unknown faces correctly recognized as unfamiliar).

Brain Area	H	BA	Ke	Ze	MNI Coordinates		
					*X*	*Y*	*Z*
PHG	R	36	56	4.31	36	-26	-21
FG	R	20	81	4.01	44	-35	-24

## Discussion

The present single case study reveals the previously undescribed neurofunctional signature of HFF as a distinct phenomenon directly related to the loss of coordinated activity between the left and right temporal lobes. In fact, while atrophy and hypofunctioning of the left temporal regions were documented both anatomically (with MRI and morphometric analysis) and functionally (with SPECT), enhanced activity in the right medial and inferior temporal cortices was uniquely associated to erroneous familiarity for unknown faces (with fMRI). Conversely, correct familiarity recognition of known faces was normal in GN and revealed a different neural signature involving the precuneus bilaterally and the right pSTS/TPJ. Activity in the precuneus is related to retrieval of episodic memories and to the acquisition of familiarity [46], while the pSTS/TPJ has a more general role in the evaluation of self-relevance, identity, and of other’s intentions [47, 48]. This pattern of activity in supramodal areas is characteristically associated to correct recognition of familiar faces in normal observers [45], with a linear increase of neural response in the precuneus and pSTS/TPJ as a function of the strength of familiarity [49].

The different activation for erroneous vs. correct familiarity recognition clearly indicates the selectivity of the right temporal cortex hyperactivity for HFF. Hence, the present neuroimaging findings are consistent with complementary evidence from prior clinical studies. In fact, while we found that hyperactivity in the right temporal cortex was associated to HFF, damage to the same area has opposite effects and leads to impaired recognition of familiar faces as well as of other types of familiar items. For example, impaired familiarity recognition with preserved recollection has been reported after lesions to the PHG [50, 51], whose hyperactivity was related to HFF in our patient. Likewise, patients with FG lesions are compromised in the identification of familiar faces [52]. Moreover, the neural response in the FG, unlike that in the pSTS/TPJ and precuneus, is typically reduced in healthy observers by the perception familiar faces [53, 54], whereas we found enhanced activity in the FG for HFF, but not for correct familiarity judgments.

The neurofunctional signature of HFF is also remarkably coherent with the behavioral performance of GN, as revealed by the signal detection analysis. In fact, GN exhibited lower discrimination sensitivity between familiar and unfamiliar faces and looser response criterion than normal participants, with a bias toward reporting face familiarity. The reduced sensitivity is likely due to a failure to use analytical strategies to encode unique facial features, which is a characteristic function of the temporal areas in the left hemisphere, in favor of a more global but less efficient encoding of facial traits implemented by the right hemisphere [3, 12]. The greater reliance on the right hemisphere therefore facilitates spurious feelings of familiarity and misattribution of personal relevance to unknown faces. These erroneous familiarity feelings cannot be counterbalanced or corrected by more precise associations in the left hemisphere between visual facial cues and specific knowledge pertaining to a unique identity and therefore lead to a liberal decision criterion concerning face familiarity recognition.

Lastly, the unusual atrophy, confined to the left temporal cortex, induced only a reduction of the functioning, rather than the more compete and impactful destruction of brain structures and functions following infarction that also leads to diaschisis and additional functional consequences in distant areas. This condition seemingly provided an ideal and unprecedented model to qualify the interhemispheric imbalance of temporal lobe functions underlying the experience of personal familiarity for unknown faces.
